# A Sparse Shared Aperture Design for Simultaneous Transmit and Receive Arrays with Beam Constraints

**DOI:** 10.3390/s23125391

**Published:** 2023-06-07

**Authors:** Dujuan Hu, Xizhang Wei, Mingcong Xie, Yanqun Tang

**Affiliations:** 1 School of Systems Science and Engineering, Sun Yat-Sen University, Guangzhou 510006, China; hudj@mail2.sysu.edu.cn; 2 School of Electronics and Communication Engineering, Sun Yat-Sen University, Shenzhen 528406, China; xiemc3@mail2.sysu.edu.cn (M.X.); tangyq8@mail.sysu.edu.cn (Y.T.)

**Keywords:** simultaneous transmit and receive, phased arrays, sparse shared aperture, beam constraints

## Abstract

The utilization of efficient digital self-interference cancellation technology enables the simultaneous transmit and receive (STAR) phased array system to meet most application requirements through STAR capabilities. However, the development of application scenario requirements makes array configuration technology for STAR phased arrays increasingly important. Thus, this paper proposes a sparse shared aperture STAR reconfigurable phased array design based on beam constraints which are achieved by a genetic algorithm. Firstly, a design scheme for transmit and receive arrays with symmetrical shared apertures is adopted to improve the aperture efficiency of both transmit and receive arrays. Then, on the basis of shared aperture, sparse array design is introduced to further reduce system complexity and hardware costs. Finally, the shape of the transmit and receive arrays is determined by constraining the side lobe level (SLL), main lobe gain, and beam width. The simulated results indicate that the SLL of the transmit and receive patterns under beam-constrained design have been reduced by 4.1 dBi and 7.1 dBi, respectively. The cost of SLL improvement is a reduction in transmit gain, receive gain, and EII of 1.9 dBi, 2.1 dBi, and 3.9 dB, respectively. When the sparsity ratio is greater than 0.78, the SLL suppression effect is also significant, and the attenuation of EII, transmit, and receive gains do not exceed 3 dB and 2 dB, respectively. Overall, the results demonstrate the effectiveness of a sparse shared aperture design based on beam constraints in producing high gain, low SLL, and low-cost transmit and receive arrays.

## 1. Introduction

The demand for digital-phased array technology has rapidly increased in various applications, including radar, communications, cognitive electronic warfare, and multifunctional integration. As a result, the importance of dynamic reconfigurable digital phased array technology has also grown significantly. Digital phased arrays usually require installing transceivers behind each antenna to achieve STAR in order to simultaneously transmit and receive signals on the same frequency [1]. STAR technology has been found to significantly enhance throughput and spectrum efficiency in wireless communication systems, as demonstrated in studies [2,3]. In addition, this technology has been applied in continuous-wave radar to achieve stealth by continuously emitting low-power waveforms that continuously illuminate the target [4,5]. Recently, STAR technology has been recognized as a disruptive technology by military radio experts, who believe that it has the potential to bring about a paradigm shift in operating on the networked electromagnetic battlefield [6,7]. To achieve STAR, it is necessary to significantly reduce the self-interference (SI) from the transmit subarray to the receive subarray at the same location; otherwise, coupled SI and noise will saturate the receiver and hinder the normal operation of the system.

A majority of studies have demonstrated that more than 50 dB of isolation can be achieved at the transmit subarray to the receive subarray of a digital phased array. Everett et al. [8] used digital transmit beamforming to reduce the self-interference received at the receive antennas in a multi-antenna system; in the case of a 72-element array partitioned as 36 transmit antennas and 36 receive antennas, 50 dB of pre-analog self-interference cancellation (SIC). Qiu et al. [9] optimized beamforming by using a linearly constrained minimum variance algorithm, achieving isolation of up to 110 dB without affecting target detection. Liang et al. [10] adopted a low-complexity precoding method to achieve adaptive transmit beamforming together with digital SIC to suppress the SI. Zhang et al. [11] explored the coupling path characteristics of all spatial links in transmit and receive arrays. They combined digital beamforming and digital SIC technology to enhance the isolation performance between the transmit aperture and array elements at different receive positions [12]. Feng Yang et al. [13] proposed a phase-only transmit beamforming method for aperture-level SIC in full duplex phased arrays. For X-band 8 × 8 transmit and receive subarrays, an average isolation improvement of about 29.3 dB was achieved with only 0.2 dB of transmit gain loss. Ao Liu et al. [14] developed a transmit SIC beamforming design to minimize the power of SI on a per-antenna basis, which reduced the need for SIC technologies other than beamforming in phased array systems. However, most of these approaches focus primarily on improving isolation using beamforming and digital cancellation techniques, with little focus on the impact of transmit and receive apertures on the STAR system.

The MIT team proposed aperture level simultaneous transmit and receive (ALSTAR) with digital beamforming and SIC technology to provide sufficient transmit-receive isolation [15]. They studied the isolation of two types of transmit and receive arrays, including left transmitting and right receiving, as well as both sides transmitting and middle receiving. The results indicated that an isolation of 163.9 dB was achieved on the 50-element phased array transmitted on the left and received on the right [16]. In 2020, they further studied the high-emission power ALSTAR system [17]. At a transmit power of 2500 W, the isolation between the transmit and receive apertures could reach 187.1 dB, while the noise floor only increased by 2.2 dB. Although the ALSTAR technology can achieve significant isolation performance, neither the transmit elements nor the receive elements in the two subarray structures they studied are fully distributed across the entire aperture.

Compared to antenna arrays that are separated into transmit and receive units, shared aperture systems can transmit and receive simultaneously within the same aperture. This significantly improves the utilization of the antenna aperture, reducing the size and cost of the system. The shared aperture design has been widely used in synthetic aperture radar, multi-frequency phased arrays, and satellite communications. Ding, Y.R. et al. [18] proposed a design of Ku/Ka-band broadband dual-polarization shared aperture antenna array with high aperture radiation efficiency. In the Ku and Ka bands, the aperture radiation efficiency can reach 86.5–96.7% and 82.1–96.9%, respectively. G. Sadhukhan et al. [19] discussed a dual-polarization S-band multi-beam shared aperture phased array antenna for ground communication and data acquisition systems. The same antenna aperture is used to simultaneously generate multiple beams for airborne detection and tracking functions. Hao, R.S. et al. [20] introduced a K-/Ka-band shared aperture end-fire phased array antenna with isolation greater than 40 dB between the K-band and Ka-band channels. Zhang, J.F. et al. [21] proposed a shared aperture phased array for K-/Ka-band satellite communication, with isolation greater than 35 dB between the K-band and Ka-band elements. Zhong, S.-S. et al. [22] presented a design of a three-frequency dual-polarization shared aperture micro-strip array antenna for synthetic aperture radar applications, with array isolation superior to 37 dB at all three frequencies.

Apart from the utilization of array aperture, the partition design of the transmit and receive arrays is also an important factor for the operation of the shared aperture phased array in the ALSTAR system. In order to reduce the performance loss caused by improper partitioning of the transmit and receive subarrays, optimization of the subarray partitioning needs to be considered. Cummings, I.T. et al. [23] proposed an information-theoretic performance metric for a narrowband STAR imaging system. They also explored how to optimally divide the array into transmit and receive apertures by applying a genetic algorithm (GA) [24]. Samaiyar et al. [25] designed the array layout of a 10 × 10 micro-strip patch antenna array, with 50 antenna elements used for transmission and another 50 antenna elements used for reception. They achieved improved transmit-receive isolation through a sparse array design. They achieved transmit-receive isolation of over 63 dB in the 27.5–28.5 GHz band, but the cost of improving the isolation was the appearance of grating lobes in the radiation pattern, and the SLL also increased. In our previous work [26], we proposed a joint design of sparse arrays and beamforming that can reduce the system cost with little performance loss of EII. Irregular and sparse array structures can often result in the high side lobe and gate lobe of the pattern. Thus, when designing the partitioning of transmit and receive arrays in a shared aperture system, it is important to ensure sufficient isolation while also optimizing the radiation pattern for low SLL. This requires careful consideration of array configurations that can achieve both goals.

This paper proposes a design for a sparse shared aperture based on beam constraints for the ALSTAR phased array system. The aim of this design is to optimize the transmit and receive subarray structure for ALSTAR while achieving sufficient SIC performance and optimizing the side lobe performance of the transmit and receive patterns. We use the 12 × 12 planar phased arrays of improved broadband microstrip antennas as an example to illustrate the specific application of sparse shared aperture design based on beam constraints for ALSTAR arrays.

The rest of the paper is given as follows. The signal model of the ALSTAR is described in Section 2. The designed ALSTAR model of the sparse shared aperture phased array based on beam constraints for ALSTAR is in Section 3 in detail. The numerical simulation results of the designed ALSTAR model are shown and analyzed in Section 4. Finally, the conclusion and future work are given in Section 5.

## 2. System Model of ALSTAR

**Figure 1:**
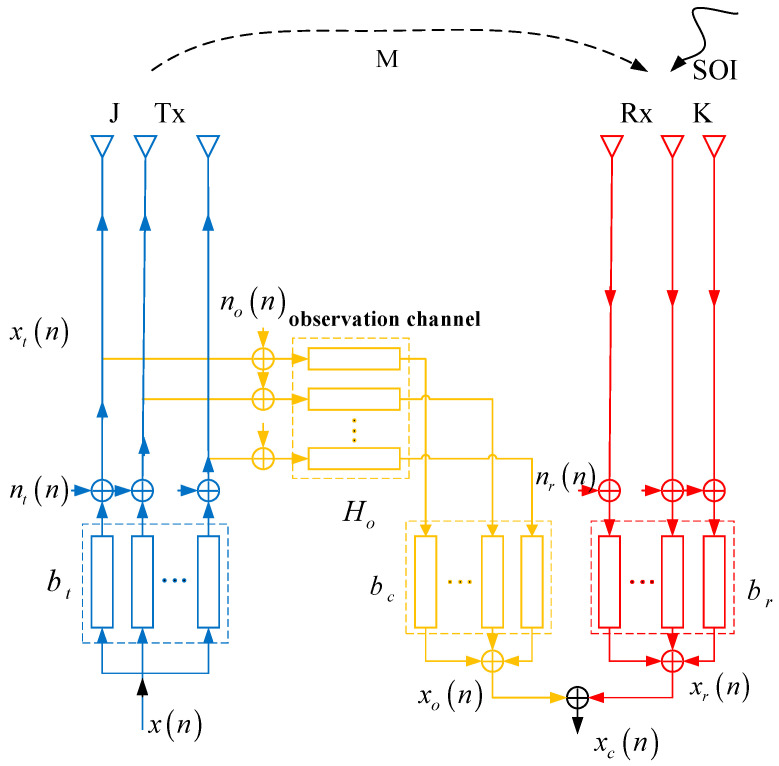
Block diagram of ALSTAR cancellation architecture.

Figure 1 outlines the digital phased array ALSTAR architecture studied in this work. We denote that there are J transmit antennas and K receive antennas in the partitioned array. The parameter $b_{t}\in {\mathbb{C}}^{J\times 1}$, $b_{r}\in {\mathbb{C}}^{K\times 1}$ and $b_{c}\in {\mathbb{C}}^{J\times 1}$ represent the vector of transmit beamforming, the vector of receive beamforming, and the adaptive cancellation filter, respectively. $M\in {\mathbb{C}}^{K\times J}$, $H_{O}\in {\mathbb{C}}^{J\times J}$ are the characteristic matrix of the coupled channel and the observation channel. When x(n)∈ℂ as the unit power $E\left[{\left|x\left(n\right)\right|}^{2}\right]=1$ to transfer the time-varying signal, the first n is a snapshot of the transmit signal $x_{t}\left(n\right)\in {\mathbb{C}}^{J\times 1}$ for

(1) $$x_{t}(n)=b_{t}\cdot x(n)+n_{t}(n),$$

where $n_{t}(n)\in {\mathbb{C}}^{J\times 1}$ represents zero-mean, additive white Gaussian noise (AWGN) due to limited dynamic range with a covariance matrix $N_{t}=E\left[n_{t}n_{t}^{H}\right]=Diag\left(b_{t}b_{t}^{H}\right)/\eta_{t}$, the symbol $\eta_{t}$ is the dynamic range of transmit channel. The Rx signal $x_{r}\left(n\right)\in {\mathbb{C}}^{K\times 1}$ after a received weight is

(2) $$x_{r}\left(n\right)=b_{r}^{H}\left[Mx_{t}\left(n\right)+s\left(n\right)+n_{r}\left(n\right)\right],$$

where the receiver noise $n_{r}\left(t\right)\in {\mathbb{C}}^{K\times 1}$ is mixed with the received signal $x_{r}\left(t\right)$. Its covariance matrix is $N_{r}=E\left[n_{r}n_{r}^{H}\right]=Diag\left(E\left[rr^{H}\right]\right)/\eta_{r}+\sigma_{r}^{2}I$, The symbol $\eta_{r}$ is the receiver dynamic range and $\sigma_{r}^{2}$ is the receiver noise power. In the ALSTAR architecture with the observation channel, the final received signal after the cancellation is given by

(3) $$x_{c}\left(n\right)=x_{r}\left(n\right)-b_{c}^{H}\left[H_{o}\left(x_{t}\left(n\right)+n_{o}\left(n\right)\right)\right],$$

By assuming $b_{c}^{H}=b_{r}^{H}MH_{o}^{-1}$ and putting that into Equation (3), $x_{c}\left(n\right)$ can be expressed as

(4) $$x_{c}\left(n\right)=b_{r}^{H}\left[n_{r}\left(n\right)+s\left(n\right)-Mn_{o}\left(n\right)\right].$$

From Equation (4), we can see that the residual signal after cancellation contains three parts, which are the receive channel noise, the observation channel noise, and the signal of interest, respectively. The signal $n_{o}\left(n\right)\in {\mathbb{C}}^{J\times 1}$ is the additive white Gaussian noise, which obeys the normal distribution $n_{o}\left(n\right)∼N\left(0,N_{o}\right)$, and $N_{o}=Diag\left(b_{t}b_{t}^{H}\right)/\eta_{r}$. According to reference [17], the EII can be formulated as

(5) $$EII=\frac{EIRP}{EIS},$$

where EIRP and EIS describe the performance of transmitter and receiver, and the $EIRP=P_{t}G_{t}$ is the effective isotropic radiated power, and the symbol $P_{t}$ and $G_{t}$ are the transmit power and the total gain of transmit array, respectively. The $EIS=P_{n}/G_{r}$ is the effective isotropic sensitivity, and the symbol $P_{n}$ and $G_{r}$ are the total residual noise power and the total gain of receive array, respectively. $P_{n}$ can be further expressed as

(6) $$\;P_{n}=b_{t}^{H}M_{bt}b_{t},$$

(7) $$\begin{array}{ll} M_{bt}= & \underset{1}{\underset{︸}{\eta_{r}^{-1}Mdiag\left(b_{r}b_{r}^{H}\right)M^{H}}}+\underset{2}{\underset{︸}{\eta_{r}^{-1}diag\left(Mb_{r}b_{r}^{H}M^{H}\right)}}\\ & +\underset{3}{\underset{︸}{\eta_{r}^{-1}\eta_{t}^{-1}diag\left(Mdiag\left(b_{r}b_{r}^{H}\right)M^{H}\right)}}+\underset{4}{\underset{︸}{\sigma_{r}^{2}/P_{t}I}}, \end{array}$$

(8) $$G_{t}\left(\phi ,\theta \right)=\left(g\left(\phi ,\theta \right)b_{t}^{H}q_{t}\left(\phi ,\theta \right)q_{t}^{H}\left(\phi ,\theta \right)b_{t}\right)/P_{t},$$

(9) $$G_{r}\left(\phi ,\theta \right)=g\left(\phi ,\theta \right)b_{r}^{H}q_{r}\left(\phi ,\theta \right)q_{r}^{H}\left(\phi ,\theta \right)b_{r}.$$

The symbol $M_{bt}$ is the covariance matrix of interference and noise. The diag(⋅) denotes the diagonal matrix, $\sigma_{r}^{2}$ denotes the noise floor, and I denotes the unit matrix of the received elements. g and $q_{t},q_{r}$ are the gain of a single element and the steering vector for the transmit and receive arrays, respectively. It is worth noting that condition ${\left\|b_{t}\right\|}^{2}=P_{t}$ must be satisfied for transmit beamforming $b_{t}$, and condition ${\left\|b_{r}\right\|}^{2}=1$ must be satisfied for receive beamforming $b_{r}$.

## 3. Sparse Shared Aperture for ALSTAR with Beam Constraints

### 3.1. Sparse Shared Aperture Design

In this paper, based on the ALSTAR system, a sparse shared aperture phased array design based on beam constraints is developed. This design has the following advantages. Firstly, when the phased array aperture is fixed, a shared aperture design is introduced to improve the aperture efficiency of the transmit array and the receive array. In this way, a narrower directional pattern of the main beam is obtained, and the detection distance and detection accuracy of the system are improved. Then, based on the shared aperture, a sparse array design is introduced to further reduce the complexity of the system and the cost of the hardware. Finally, an objective function is constructed using SLL, main beam gain, and beam width to constrain the pattern of the transmit and receive arrays. It is also possible to reduce SLL while maintaining high transmit and receive gains in desired directions. GA is a well-studied method of solving the issue of sidelobe suppression [27], array partitioning [28], and sparse arrays [29,30]. Therefore, this paper applies GA to the uniform planar phased array ALSTAR system to design shared aperture transmit and receive subarray under beam constraints. The number of individuals in GA is NP, The number of genes in an individual is D. In a planar phased array, D also represents the total number of transmit array elements and receive array elements. So a single individual can be expressed as

(10) $$ff_{i}=\left(f_{1},f_{2},\cdots ,f_{D}\right),i\in \left(1,2,\cdots ,NP\right),$$

where i represents the serial number of the individual in the population. The irregular subarray approach reduces peak sidelobes by breaking up the subarray phase center periodicity but still results in very high average sidelobes and directivity loss. Therefore, we consider constructing a symmetric transmit subarray and receiving subarray on a uniform planar array. In other words, $ff_{i}$ needs to meet the requirement that the transmit and receive arrays are symmetrically distributed up and down and left and right. For example, first, construct a fundamental matrix A with 2 rows and 3 columns

(11) $$A=\left[\begin{array}{ccc} 0 & -1 & 1\\ -1 & 1 & 0 \end{array}\right].$$

Each element in the matrix A represents an element in the plane array. According to the fundamental matrix A, the matrix B with four rows and six columns is obtained that is symmetric up and down and left and right. The premise is that the number of rows and columns of matrix B is an even multiple of the number of rows and columns of matrix A. The matrix B can be represented by Equation (12).

(12) $$B=\left[\begin{array}{cccccc} 0 & -1 & 1 & 1 & -1 & 0\\ -1 & 1 & 0 & 0 & 1 & -1\\ -1 & 1 & 0 & 0 & 1 & -1\\ 0 & -1 & 1 & 1 & -1 & 0 \end{array}\right].$$

When the number of rows and columns of the symmetric matrix B is M and N, each individual is a two-dimensional binary value matrix with the number of genes D=M×N. Individual $ff_{i}$ can be obtained by rearranging the matrix B into a one-dimensional column vector. Where 1 means transmit array element and 0 means receive array element. −1 indicates that the array element at this position is not working. One-half of the number of −1 is the number of sparse transmit and receive elements, recorded as $N_{sparse}$. The sparsity rates $sparse_{rate}$ of the transmit and receive arrays are as follows

(13) $$sparse_{rate}=\left(J(K)-N_{sparse}\right)/J(K).$$

### 3.2. Optimization Model Design

In a uniform planar phased array antenna, the desired direction of the array pattern is (ϕ,θ), then the steering vector $q_{SSA}$ for transmit and receive array elements under sparse shared aperture can be expressed as

(14) $$q_{SSA}\left(\phi ,\theta \right)=exp\left[-j\frac{2\pi }{\lambda }\left(\vec{x}cos\left(\phi \right)sin\left(\theta \right)+\vec{y}sin\left(\phi \right)sin\left(\theta \right)\right)\right].*ff_{i},$$

(15) $$\{\begin{array}{l} q_{t\_SSA},\;\;\;\;\;\;\;\;ff_{i}|f_{D}=1\\ q_{r\_SSA},\;\;\;\;\;\;\;\;ff_{i}|f_{D}=0\\ q_{SSA\_sparse},\;\;ff_{i}|f_{D}=-1 \end{array}.$$

where $\vec{x}$ represents the distance from each element in the array plane to the x-axis, $\vec{y}$ represents the distance from each element in the array plane to the y-axis. After sparse shared aperture design, the steering vectors of transmit and receive arrays are represented by $q_{SSA\_t}$ and $q_{SSA\_r}$, respectively. Therefore, the transmit pattern $G\_SSA_{t}\left(\phi ,\theta \right)$ or receive pattern $G\_SSA_{r}\left(\phi ,\theta \right)$ for the desired direction (ϕ,θ) in a planar array can be expressed as

(16) $$G\_SSA_{t}\left(\phi ,\theta \right)=\left(g_{t\_SSA}\left(\phi ,\theta \right)\cdot *\;b_{t\_SSA}^{H}q_{t\_SSA}\left(\phi ,\theta \right)q_{t\_SSA}^{H}\left(\phi ,\theta \right)b_{t\_SSA}\right)/P_{t},$$

(17) $$G\_SSA_{r}\left(\phi ,\theta \right)=g_{r\_SSA}\left(\phi ,\theta \right)\cdot *\;b_{r\_SSA}^{H}q_{r\_SSA}\left(\phi ,\theta \right)q_{r\_SSA}^{H}\left(\phi ,\theta \right)b_{r\_SSA},$$

(18) $$\begin{array}{l} M_{bt\_SSA}=\underset{1}{\underset{︸}{\eta_{r}^{-1}M_{SSA}diag\left(b_{r\_SSA}b_{r\_SSA}^{H}\right)M_{SSA}^{H}}}+\underset{2}{\underset{︸}{\eta_{r}^{-1}diag\left(M_{SSA}b_{r\_SSA}b_{r\_SSA}^{H}M_{SSA}^{H}\right)}}\\ \;\;\;\;\;\;\;\;\;+\underset{3}{\underset{︸}{\eta_{r}^{-1}\eta_{t}^{-1}diag\left(M_{SSA}diag\left(b_{r\_SSA}b_{r\_SSA}^{H}\right)M_{SSA}^{H}\right)}}+\underset{4}{\underset{︸}{\sigma_{r}^{2}/P_{t}I}}, \end{array}$$

(19) $$\;P_{n\_SSA}=b_{t\_SSA}^{H}M_{bt\_SSA}b_{t\_SSA},$$

(20) $$EII_{SSA}=\frac{EIRP_{SSA}}{EIS_{SSA}}=\frac{P_{t}*G\_SSA_{t}}{P_{SSA\_n}/G\_SSA_{r}}=\frac{P_{t}}{P_{n\_SSA}}G\_SSA_{t}*G\_SSA_{r},$$

where, $M_{bt\_SSA}$ and $P_{n\_SSA}$ denote the covariance matrix and power of the residual noise with sparse shared aperture design, respectively. Moreover, the method for obtaining the coupling matrix $M_{SSA}$ in the Equation (18) refers to the extraction method in the reference [31]. The EII, EIRP, EIS, and $P_{n}$ of ALSTAR systems with sparse shared aperture design are represented as $EII_{SSA}$, $EIRP_{SSA}$, $EIS_{SSA}$, and $P_{n\_SSA}$, respectively.

**Figure 2:**
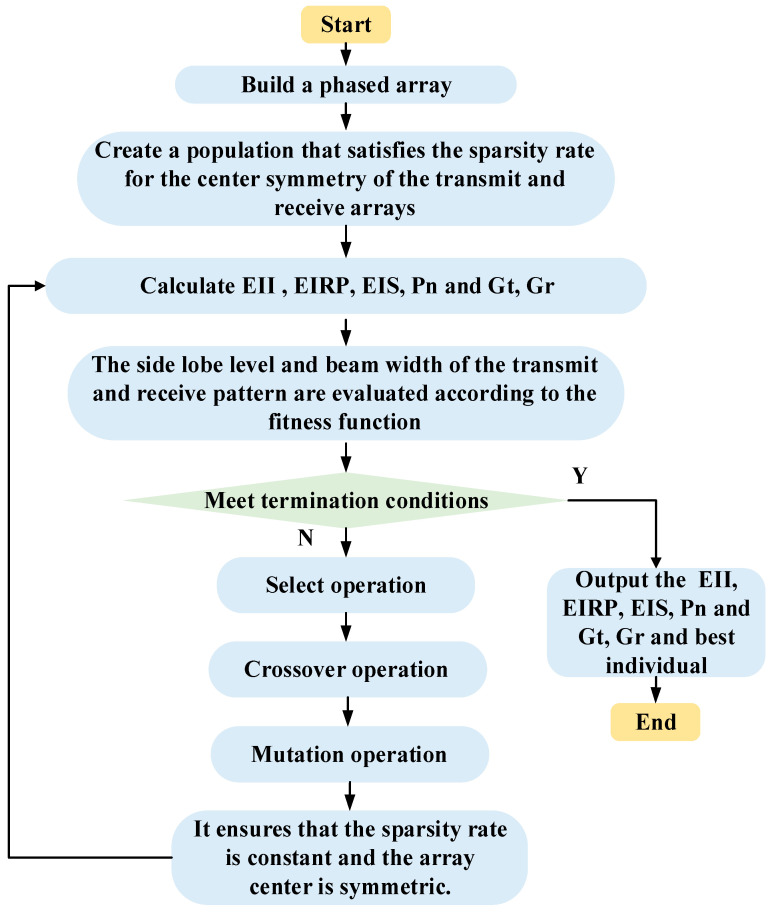
The flowchart of sparse shared aperture design based on beam constraints.

According to the design of a sparse shared aperture, it is necessary to further investigate whether the transmit and receive patterns in this configuration meet the desired pattern through beam constraints. It is more direct in a physical sense to use the pattern of transmit and receive subarray to construct fitness functions. Therefore, the SLL, main lobe gain, and beam width of the pattern can be constrained within a certain range to obtain the desired transmit and receive pattern. Here, the desired SLL is defined as $G_{t/r}{}_{{}_{SLL_{de}}}$, the desired main lobe gain as $G_{t/r}{}_{{}_{MLG_{de}}}$, and the desired beam width as $G_{t/r}{}_{{}_{BW_{de}}}$. The optimized SLL, main beam gain, and beam width can be denoted as $G\_SSA_{t/r}{}_{{}_{SLL}}$, $G\_SSA_{t/r}{}_{{}_{MLG}}$ and $G\_SSA_{t/r}{}_{{}_{BW}}$, respectively. Therefore, the error difference among the three can be expressed by

(21) $$\{\begin{array}{c} \Delta_{SLL}=G\_SSA_{t/r}{}_{{}_{SLL}}-G_{t/r}{}_{{}_{SLL_{de}}}\\ \Delta_{MLG}=G\_SSA_{t/r_{MLG}}-G_{t/r}{}_{{}_{MLG_{de}}}\\ \Delta_{BW}=G\_SSA_{t/r}{}_{{}_{BW}}-G_{t/r}{}_{{}_{ND_{BW}}} \end{array},$$

where $\Delta_{SLL}$, $\Delta_{MLG}$ and $\Delta_{BW}$ represent the difference between the optimal value and the expected value of the SLL, main lobe gain, and beam width, respectively. Thus, the objective function or fitness function complying with the above conditions can be provided as

(22) $$fitness=K_{SLL}\Delta_{SLL}^{2}+K_{MLG}\Delta_{MLG}^{2}+K_{BW}\Delta_{BW}^{2},$$

where the fitness signifies the total objective function value, which consists of three parts, as shown in Equation (22). The coefficient $K_{SLL}$, $K_{MLG}$ and $K_{BW}$ indicate the weight of each part, and $K_{SLL}+K_{MLG}+K_{BW}=1$. The interaction of the three goals can be achieved by modifying their weights. By setting different weights and aiming to minimize the fitness value to obtain the desired transmit pattern and receive pattern. The specific flow of sparse shared aperture design based on beam constraints is shown in Figure 2. The specific optimization process is as follows.

1. First, initialize a binary population in which all individuals are symmetrical up and down, left and right.
2. Then, calculate the fitness value of each individual according to Equation (22), get the individual with the best fitness value, and judge whether it meets the termination criterion.
3. If it is satisfied, the algorithm stops, and the optimal individual is output as the optimization result; if not, the genetic operation of selection, crossover, and mutation is performed on the individuals in the population.
4. Finally, ensure that the geometric shapes of the transmit and receive array formed after each individual rearrangement in the newly generated population are symmetrical up and down, left and right.
5. For the evolved offspring population, the termination criterion is judged again, and the cycle proceeds until the termination condition is satisfied.

## 4. Simulation Results

In order to analyze the impact of the sparse shared aperture design based on beam constraints on the isolation of the ALSTAR system, as well as the impact on the pattern of the transmit array and receive array. Due to the direct correlation between the coupling matrix and element gain of the ALSTAR array and the isolation of the system, we designed a 12 × 12 uniform planar phased array with high isolation and gain for the ALSTAR system. Subsequently, the coupling matrix and element gain data of the phased array were extracted, and the performance of the sparse shared aperture ALSTAR array based on beam constraints was discussed and analyzed based on the extracted data. Finally, we explore shared aperture transmit and receive array configurations under beam constraints with different weights and shared aperture transmit and receive array configurations with beam constraints with fixed weights and different sparse rates.

### 4.1. Phased Array Design

**Figure 3:**
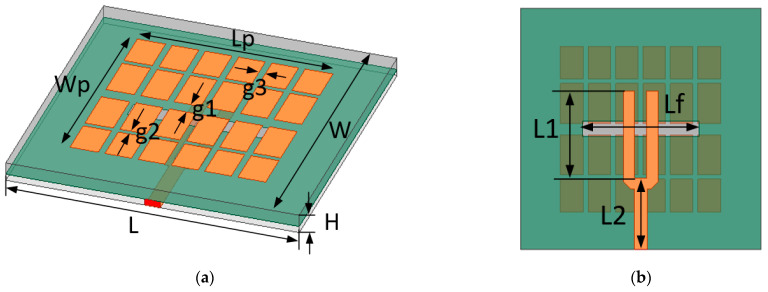
The structure of the improved wideband microstrip antenna element: (**a**) the front of the antenna, (**b**) The back of the antenna.

In this subsection, an example is provided to assess the effectiveness of the proposed method by the design of an improved wideband microstrip antenna based on microstrip antenna, and its structures are shown in Figure 3. The antenna unit is composed of double-layer dielectric substrates, one of which is used for radiation, and the other is used for feeding. The material of the two dielectric substrates is Rogers 4350B, its dielectric constant is 3.48, the thickness of the upper substrate is 1.524 mm, the thickness of the lower substrate is 0.813 mm, and the loss tangent of the substrate material is 0.001. Traditional microstrip antennas have narrow bandwidths, which are difficult to meet the requirements of wideband X-band radars. In order to achieve a large impedance bandwidth, a broadband high-gain planar antenna is designed. The radiation patch of the antenna is composed of several small patches similar to “mushrooms” of different sizes. These patches are coupled together to form metasurface-like properties with extended bandwidth. The antenna is fed by means of aperture coupling, and the fed microstrip line is designed in shape similar to a “fork”, which is beneficial to realize broadband matching. When the feed slot is just below the central gap between the mushroom patches, multiple resonant modes are excited simultaneously, thereby broadening the impedance bandwidth of the antenna. Table 1 lists the dimension parameters of the antenna.

**Figure 4:**
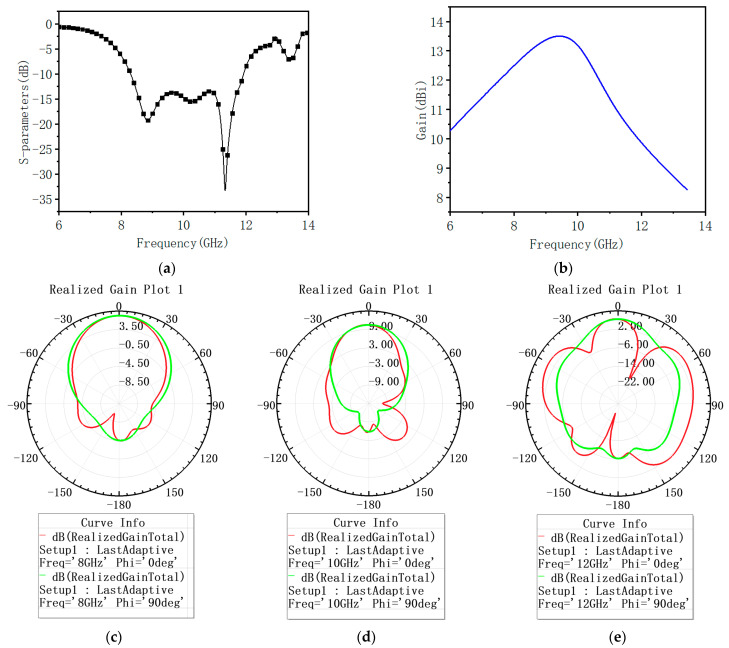
The performance of improved wideband antenna element: (**a**) The S parameter of the antenna at a frequency of 8–12 GHz; (**b**) the gain of the antenna at a frequency of 8–12 GHz; (**c**) the pattern of the antenna at a frequency of 8 GHz; (**d**) the pattern of the antenna at a frequency of 10 GHz; (**e**) the pattern of the antenna at a frequency of 12 GHz.

**Figure 5:**
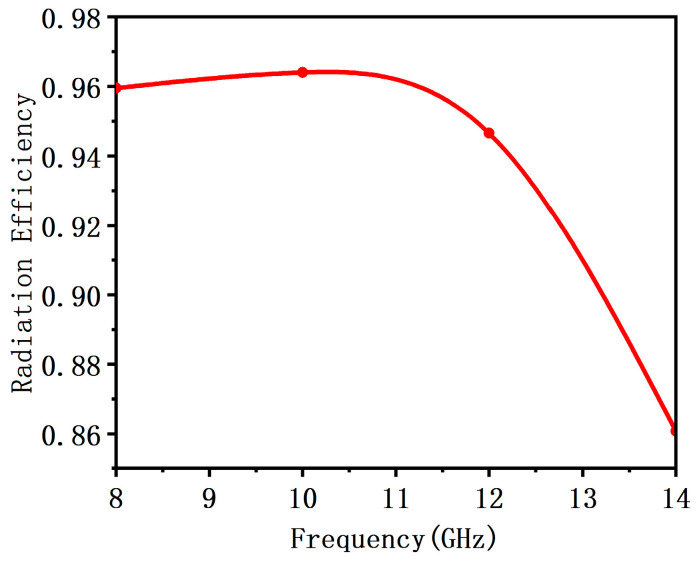
The characteristic parameters of improved wideband antenna element.

Using HFSS software to simulate the proposed antenna unit and the simulation results are shown in Figure 4 and Figure 5. It can be seen that the bandwidth of the proposed antenna unit, whose reflection coefficient is less than −10 dB is 8–12 GHz, and within this bandwidth range, the gain of the antenna is greater than 9 dBi. The antenna can maintain a good and stable radiation pattern in the 8–10 GHz frequency band. Although the SLL of the pattern increase at 12 GHz, the maximum radiation direction remains directly above. In addition, within the working bandwidth, the radiation efficiency of the antenna is above 84%.

**Figure 6:**
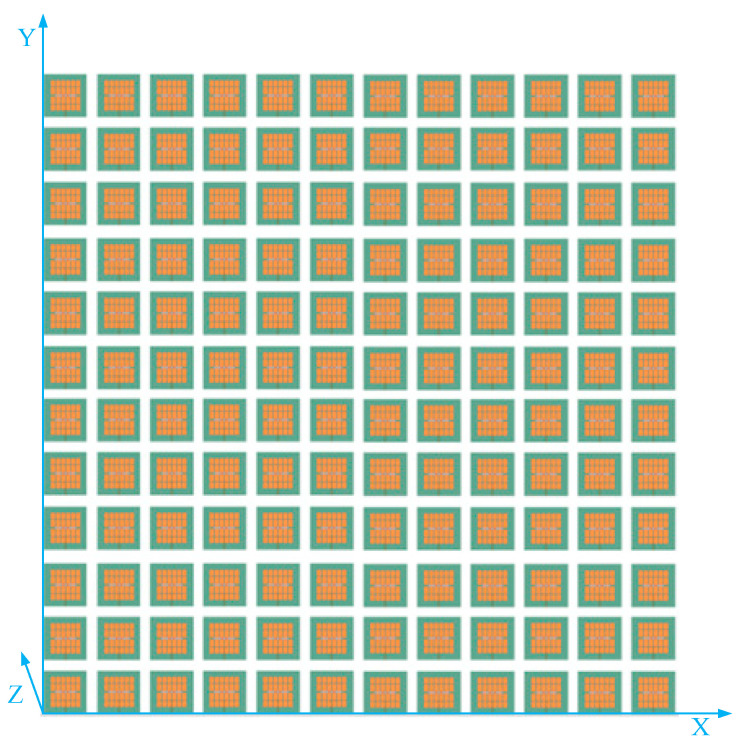
Schematic diagram of the 12 × 12 array structure.

Using the antenna above as the array element, design a 12 × 12 large-scale antenna array. The structure of the antenna array is shown in Figure 6. The array elements are evenly spaced in the X-axis and Y-axis directions. Using the method described in reference [27] to extract the gain pattern of each array element and the coupling matrix M between them, bring the data into Equation (7) for simulation.

### 4.2. Performance Analyze with Sparse Shared Aperture ALSTAR Arrays

In order to reveal the effect of ALSTAR array with sparse shared aperture based on beam constraints, we conduct two simulations using shared aperture design and sparse shared aperture design and analyze the performance of ALSTAR under different weights and sparsity rates. The ALSTAR array adopts the planar phased array designed above, with a beam scanning range of ± 70°. The dynamic range of the transmit channel is 45 dB, and the dynamic range of the receive channel is 70 dB. The receive channels have thermal noise power of −91 dBm, which is obtained by the 100 MHz bandwidth channel with a 3 dB noise figure. We provide results with a transmit power of 30 w, a GA population size of 100, and an iteration count of 200. Conduct 200 experiments independently using MATLAB software (Version: R2021a) to ensure the reliability of the experimental results. The experiment was conducted on a desktop PC with an Intel Core i7-8700 CPU processor @ 3.20 GHz, 16 GB RAM, under the Windows 10 64-bit OS.

#### 4.2.1. Shared Aperture Design

The first part simulates the case of shared aperture design with beam constraints, and several numerical results are presented to demonstrate the benefits of the proposed design. The side lobe level, main beam gain, and beam width of the pattern are given different weights, and it is beneficial to dynamically trade them off. In order to better demonstrate the sparse shared aperture design based on beam constraints under different weights. Three weight settings are considered in the following simulations. The three cases are $K_{SLL}=0.5,\;K_{MLG}=0.4,\;K_{BW}=0.1$, $K_{SLL}=0.4,\;K_{MLG}=0.5,\;K_{BW}=0.1$, and $K_{SLL}=0.3,\;K_{MLG}=0.3,\;K_{BW}=0.4$.

**Figure 7:**
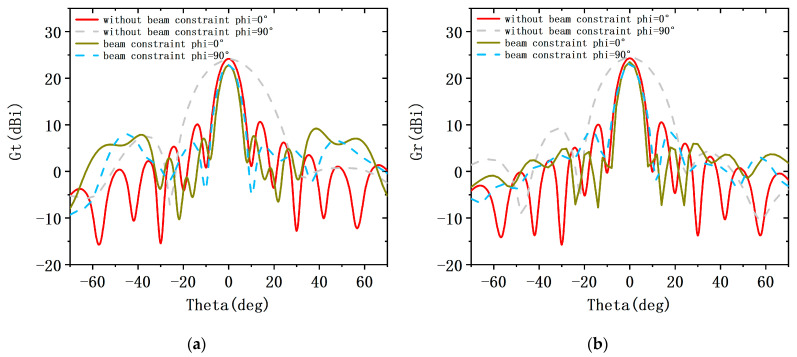
Pattern of the conventional ALSTAR array along with that of the desiged array architecture at the $K_{SLL}=0.5,\;K_{MLG}=0.4,\;K_{BW}=0.1$. (**a**) the transmit pattern $G_{t}$, (**b**) the receive pattern $G_{r}$.

**Figure 8:**
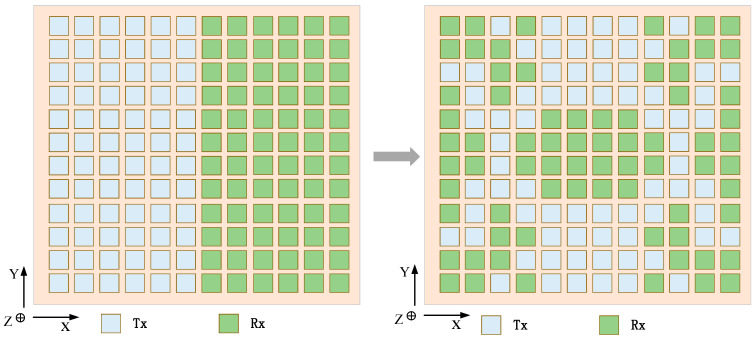
Array architectures for ALSTAR: (**left**) convention array, and (**right**) desiged array architectures at the $K_{SLL}=0.5,\;K_{MLG}=0.4,\;K_{BW}=0.1$.

In the first case: the weight of the fitness function is set to $K_{SLL}=0.5,\;K_{MLG}=0.4,\;K_{BW}=0.1$. Figure 7 shows the transmit and receive patterns of traditional arrays without beam constraints and shared aperture arrays with beam constraints. When the beam is scanned to 0°, the pattern is compared with phi = 0°. The transmit and receive patterns without beam constraints have main lobe gains of 23.38 dBi and 23.49 dBi, SLL of 10.78 dBi and 10.68 dBi, and beam widths of 8°. Although the attenuation of the main lobe gain after beam constraints is less than 2 dBi, the SLL is increased by 5.65 dBi and 7.2 dBi, respectively. However, there has been no significant improvement in beam width. Then, comparing the pattern with phi = 90°, it can be seen that the main lobe width of the transmit pattern with beam constraint is almost half that without beam constraints because the aperture utilization of the transmit and receive arrays in the shared aperture is twice as high as that of traditional arrays. Under beam constraints, the side lobe level also decreases to a certain extent. Furthermore, the transmit and receive subarrays corresponding to the beam-constrained transceiver pattern are as shown in the left figure in Figure 8. The receive and transmit subarrays share the aperture, and the shape of the array is symmetrical from top to bottom and from left to right.

**Figure 9:**
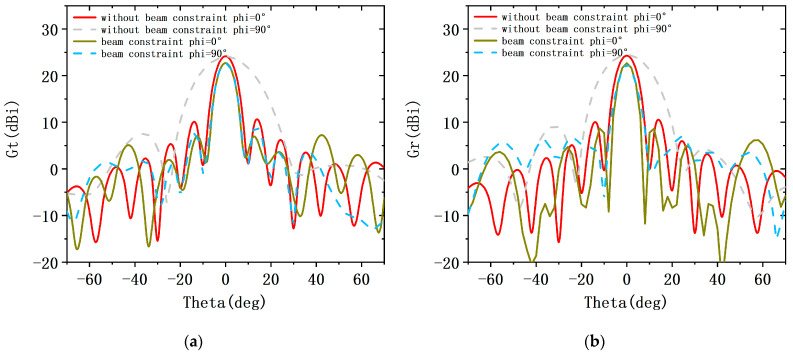
Pattern of the conventional ALSTAR array along with that of the desiged array architecture at the $K_{SLL}=0.4,\;K_{MLG}=0.5,\;K_{BW}=0.1$. (**a**) the transmit pattern $G_{t}$, (**b**) the receive pattern $G_{r}$.

**Figure 10:**
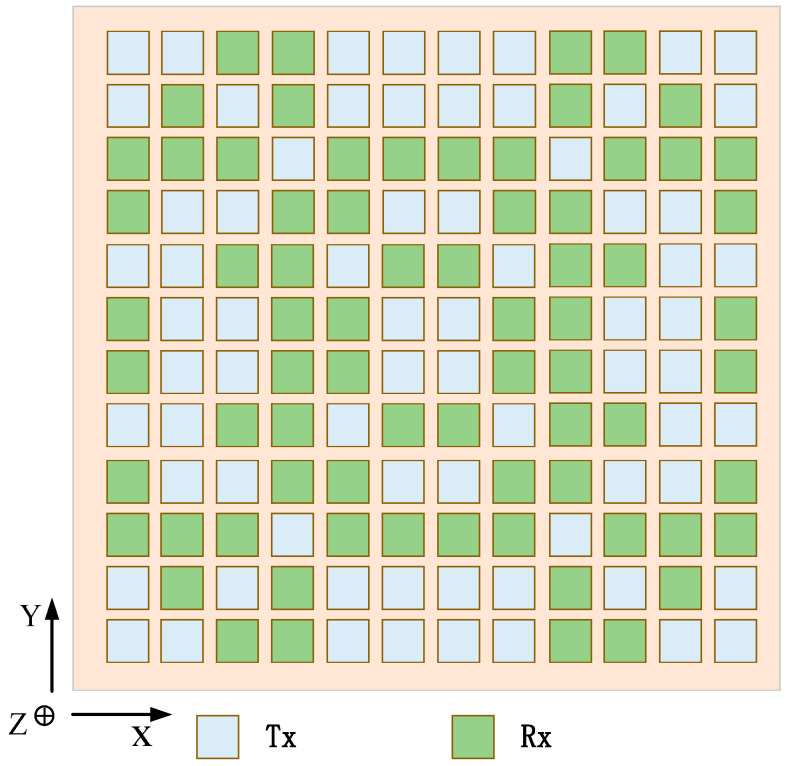
Designed array architecture for ALSTAR at the $K_{SLL}=0.4,\;K_{MLG}=0.5,\;K_{BW}=0.1$.

In the second case, the weight of the fitness function is set to $K_{SLL}=0.4,\;K_{MLG}=0.5,\;K_{BW}=0.1$. Figure 9 shows the transmission and reception patterns of traditional arrays without beam constraints and shared aperture arrays with beam constraints. When the beam is scanned to 0°, the pattern is compared with phi = 0°. After beam constraints, the SLL and beam width of the receive and transmit pattern have significantly improved, and the attenuation of the main lobe gain does not exceed 1 dB. However, serious grating lobes appear in the emission pattern. The SLL is slightly suppressed at phi = 90°. Figure 10 shows the transmit and receive arrays corresponding to the transmit and receive patterns under beam constraints. Compared to the transmit and receive subarrays obtained under the previous constraint, the aperture of the transmit and receive array in this configuration is relatively large, so the beam width of the pattern is also relatively narrow.

**Figure 11:**
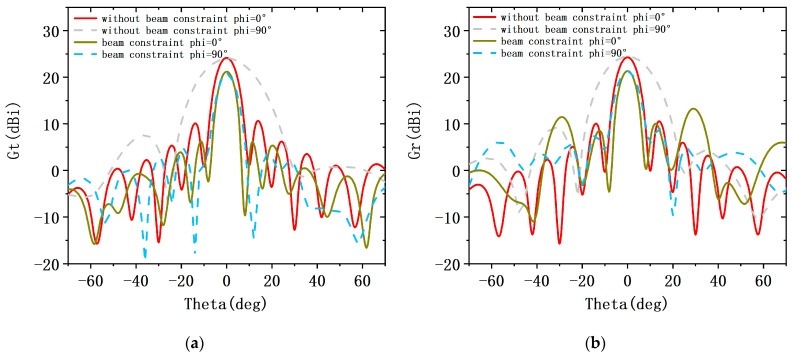
Pattern of the conventional ALSTAR array along with that of the desiged array architecture at the $K_{SLL}=0.3,\;K_{MLG}=0.3,\;K_{BW}=0.4$. (**a**) the transmit pattern $G_{t}$, (**b**) the receive pattern $G_{r}$.

**Figure 12:**
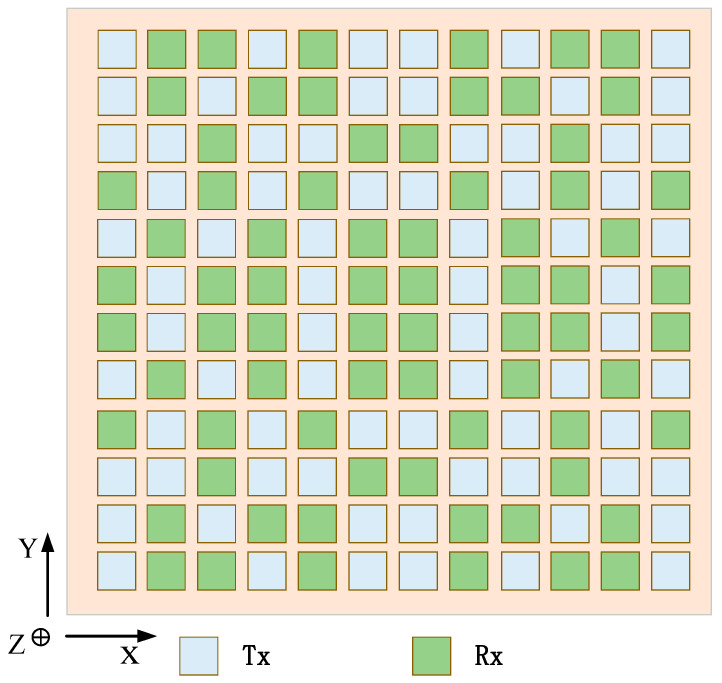
Desiged array architecture for ALSTAR at the $K_{SLL}=0.3,\;K_{MLG}=0.3,\;K_{BW}=0.4$.

In the third case, the weight of the fitness function is set to $K_{SLL}=0.3,\;K_{MLG}=0.3,\;K_{BW}=0.4$. As seen the Figure 11, the SLL, main lobe gain, and beam width have significant reduction advantages in the designed array, but peak gain decreases significantly. Although the beam width of the received pattern also exhibits a reduction advantage, the SLL does not decrease, the attenuation of the main lobe gain reaches 3 dBi, and there is also a phenomenon of gate lobe protrusion. The SLL is significantly suppressed at phi = 90°. By observing Figure 12, it is found that the transmit and receive arrays obtained under this beam constraint are also arranged with transmit and receive elements within the entire aperture, so the main beam of the obtained receive and transmit pattern will be narrower than the main beam of the left transmitting and right receiving pattern.

#### 4.2.2. Sparse Shared Aperture Design

**Figure 13:**
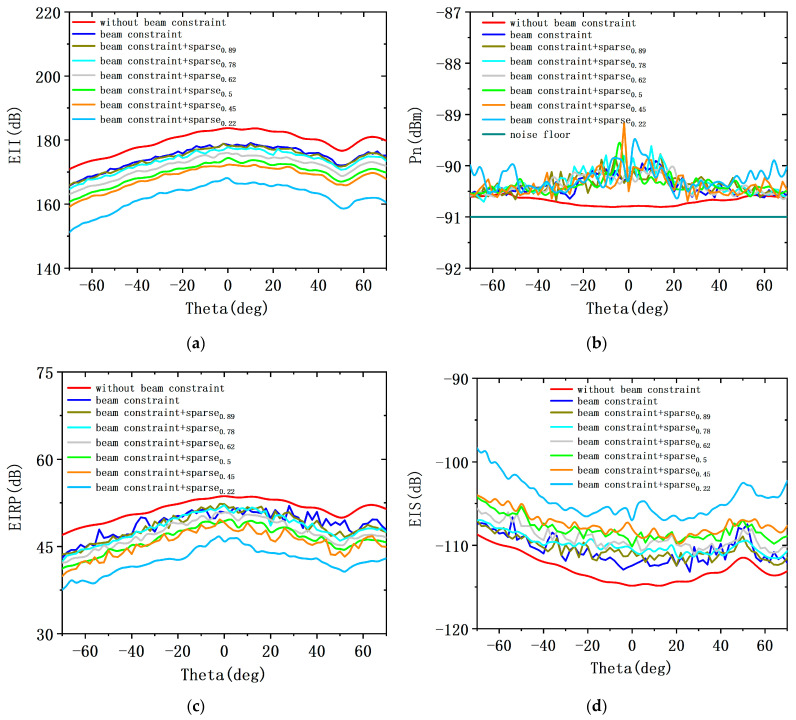

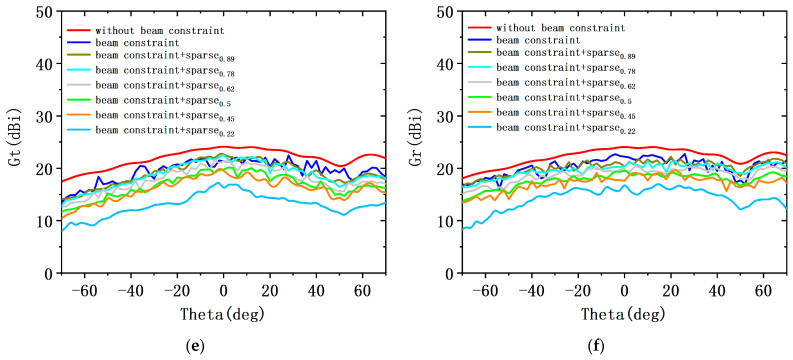
Comparison of performance indicators of the system under sparse rates: (**a**) the EII of ALSTAR system; (**b**) the $P_{n}$ of ALSTAR system; (**c**) the EIRP of ALSTAR system; (**d**) the EIS of ALSTAR system; (**e**) the $G_{t}$ of ALSTAR system; (**f**) the $G_{r}$ of ALSTAR system.

The second part simulates the case of sparse shared aperture design with beam constraints. According to the comparison and analysis of the three beam constraints in the previous section, setting the weight value to $K_{SLL}=0.5,\;K_{MLG}=0.4,\;K_{BW}=0.1$ can achieve better comprehensive performance. Therefore, this section further analyzes the array performance of ALSTAR with sparse shared aperture design; Figure 13 shows the performance realized across scan angles without beam constraints, shared aperture based on beam constraints, and sparse shared aperture based on beam constraints with 30 W of transmit power. The shared aperture design achieves EII 179.68 dB at beam scan angles (out to 30°), with an average isolation reduction of 4 dB compared to the without beam constraints. At the sparsity rate of 0.89 and the scan angle of 0°, the EII of sparse shared aperture and the EII of shared aperture are almost the same. The sparsity rate of 0.89 means that the cost of the ALSTAR array is reduced by 11%.

In addition, Figure 13a also shows the EII achieved across scan angles for the sparse shared aperture design at different sparsity rates. Note that the EII decreases rapidly at the sparsity rate below 0.45 and reduces slowly at the sparsity rate above 0.45. Figure 13b–f show the noise power $P_{n}$, the EIRP, the EIS, transmit total gain $G_{t}$, and receive total gain $G_{r}$ achieved across scan angles. Except for $P_{n}$, the changing trend of the EIRP, EIS, $G_{t}$, and $G_{r}$ is similar to that of EII under different sparsity rates. At the scan angle of 0°, the $P_{n}$ of shared aperture and sparse shared aperture is only 0.9 dB and 1.2 dB above the thermal noise floor, respectively. Within 30° of broadside, the reduction in $G_{t}$ and $G_{r}$ of the sparse shared aperture with a sparsity rate of 0.89 is 1.9 dBi and 2.1 dBi compared to the without beam constraints, respectively.

**Figure 14:**
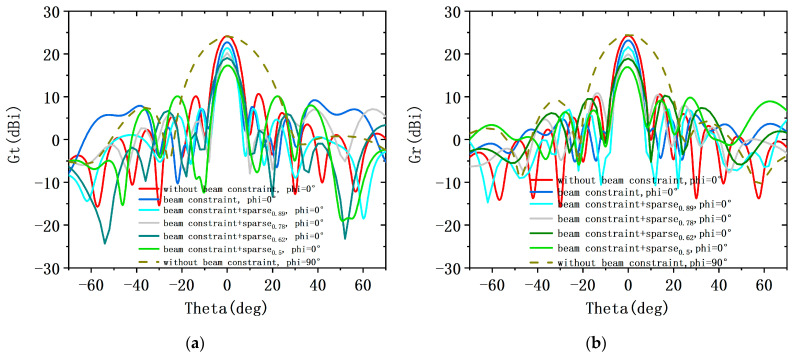
The $G_{t}$ and $G_{r}$ curves of without beam constraint, shared aperture design and sparse shared aperture design at four sparsity rates in different scan angle: (**a**) the $G_{t}$ of ALSTAR system; (**b**) the $G_{r}$ of ALSTAR system.

**Figure 15:**
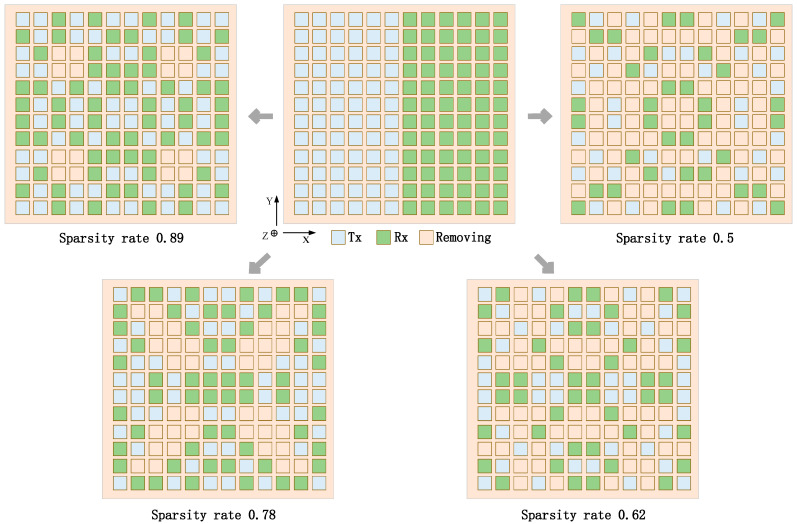
Conventional arrays and sparse shared aperture arrays configuration at four sparsity rates.

The far-field transmit and receive patterns of sparse shared aperture based on beam constraints at sparsity rates of 0.89, 0.78, 0.62, and 0.5 are compared to the without beam constraint and shared aperture based on beam constraints, as shown in Figure 6. At the beam scan angle of 0°, Figure 14a,b show that the peak directivity of transmit pattern and receive pattern for the three cases remains similar. Compared to the $G_{t}$ with a shared aperture design, the main lobe gain of the $G_{t}$ with a sparse shared aperture design is reduced by 1.4 dBi, 2.1 dBi, 3.9 dBi, and 4.8 dBi at sparsity ratios of 0.89, 0.78, 0.62, and 0.5, respectively. Moreover, the main lobe gain $G_{r}$ is also reduced by 1.9 dBi, 2.9 dBi, 4.3 dBi, and 5.9 dBi, respectively. Obviously, the efficiency of the sparse transmit array aperture and the efficiency of the sparse receive array aperture remain consistent. In addition, the side lobe suppression of the transmit patterns is relatively significant at a sparse rate of 0.89, 0.78, and 0.62, and the pattern has significant gate lobes at a sparse rate of 0.78. However, the transmit pattern exhibits undesirable side lobe suppression at sparsity rates of 0.5. Therefore, when the sparsity ratio exceeds less than 0.62, it is difficult to obtain an ideal transmit and receive pattern under beam constraints. Furthermore, Figure 15 shows the array configuration achieved under different sparsity rates. The sparsity rate is 0.89, 0.78, 0.62, and 0.5, respectively. Sparse shared aperture design based on beam constraints can achieve the design of shared aperture for both transmit and receive arrays with fewer transmit and receive elements. Moreover, the side lobe of the directional pattern of the designed transmit and receive arrays has a significant suppression effect, and the attenuation of the main lobe gain does not exceed 2 dB. It is further proved that this design enables the ALSTAR system to achieve a good comprehensive performance.

## 5. Conclusions and Future Work

In this paper, we propose the sparse shared aperture design based on beam constraints for the ALSTAR phased arrays. The sparse shared aperture design is introduced to reduce the cost of the array and improve the utilization of the transmit and receive array apertures. The isolation performance and pattern of ALSTAR are traded off by constraining the SLL, main lobe gain, and beam width of the transmit and receive pattern. A 12 × 12-phased array of improved broadband micro-strip antennas is used to verify the effectiveness of the proposed design. The simulation results show that compared with the traditional transmit and receive array structure, the transmit and receive array based on the beam-constrained sparse shared aperture design can obtain low side lobe at a sparse rate greater than 0.62, and the EII and main lobe gains of ALSTAR are only slightly decline. In our future work, the proposed sparse shared aperture design will be applied in different scenarios to further verify its effectiveness.

## Figures and Tables

**Table 1:** Size Parameters of Antenna Unit (Unit: mm).

Parameter	Value
Wp	23.5
W	35
L1	10.5
L2	13.2
Lp	24.3
L	35
Lf	17
g1	1.63
g2	0.3
g3	0.5
H	2.337

## Data Availability

Not applicable.
